# Association between cemented and uncemented cups and risk of revision in patients aged 65 years or older following primary total hip arthroplasty: a study of 310,515 patients from the Nordic Arthroplasty Register Association

**DOI:** 10.2340/17453674.2026.46937

**Published:** 2026-10-28

**Authors:** Roshan LATIFI, Simon KORNVIG, Maziar MOHADDES, Muhanned ALI, Torbjørn Berge KRISTENSEN, Geir HALLAN, Antti ESKELINEN, Keijo MÄKELÄ, Claus VARNUM, Søren OVERGAARD

**Affiliations:** 1 Department of Orthopedic Surgery, Aalborg University Hospital, Denmark; 2 Department of Orthopedic Surgery, Lillebaelt Hospital – Vejle, Denmark; 3 Department of Regional Health Research, University of Southern Denmark, Denmark; 4 Department of Orthopedics, Institute of Clinical Sciences, The Sahlgrenska Academy, University of Gothenburg, Gothenburg, Sweden; 5 Department of Orthopedics, Skåne University Hospital, Hässleholm, Sweden and Department of Clinical Sciences – Orthopedics, Lund University, Lund, Sweden; 6 Unit of Orthopedics, Department of Clinical Sciences, Malmö, Lund University, Sweden; 7 The Norwegian Arthroplasty Register, Department of Orthopedic Surgery, Haukeland University Hospital, Bergen, Norway; 8 Department of Clinical Medicine, University of Bergen, Norway; 9 Coxa Hospital for Joint Replacement, and Faculty of Medicine and Health Technologies, University of Tampere, Tampere, Finland; 10 Turku University Hospital and University of Turku, Finland, and the Finnish Arthroplasty Register; 11 Copenhagen University Hospital Bispebjerg, Department of Orthopedic Surgery and Traumatology and Department of Clinical Medicine, University of Copenhagen, Denmark

## Abstract

**Background and purpose:**

Cemented stem fixation in total hip arthroplasty (THA) reduces early femoral fracture risk in elderly patients, but the optimal method of cup fixation remains controversial. We aimed to compare cup revision risk between cemented and uncemented acetabular cups in patients aged 65 years or older undergoing primary THA.

**Methods:**

This registry-based cohort study used data from the Nordic Arthroplasty Register Association (NARA). We included patients aged 65 years or older who underwent primary THA for primary osteoarthritis or sequelae of childhood hip disorders between 1995 and 2018. The primary outcome was all-cause cup revision, with prespecified analyses stratified by age and sex. Cox regression adjusted for age, sex, femoral head size, time period, and nation was used. Follow-up was divided into 3 periods: 0–1, 1–6, and 6–26 years.

**Results:**

Among 310,515 patients, cemented cups were associated with a lower risk of all-cause cup revision during the first year (adjusted hazard ratio [aHR] 0.47, 95% confidence interval [CI] 0.41–0.53) and a higher risk at 6–26 years (aHR 1.91, CI 1.72–2.13). In patients aged 80 years or older, the sex-specific adjusted hazard ratios after the first year were close to 1, with wide confidence intervals.

**Conclusion:**

Cemented cups were associated with a lower risk of cup revision during the first year across all age groups and a higher risk at 6–26 years in the overall cohort. Among patients aged 80 years or older, we found no evidence of an association between fixation method and cup revision after the first year.

In elderly patients, osteoporosis and low bone mineral density are associated with a higher risk of periprosthetic fracture, aseptic loosening, and revision after THA, and these factors should be considered when choosing femoral fixation [1]. Cemented femoral fixation has been associated with a reduced risk of revision due to periprosthetic femoral fracture in women aged 75 years or older [2,3].

The acetabular component has been less extensively analyzed, as previous studies have frequently focused on the entire THA [4–6].

Conflicting data exists regarding overall and cause-specific revision rates of different acetabular cup fixation methods [7,8]. Despite the increasing use of uncemented acetabular components, evidence specifically addressing elderly patients is limited [9], representing a significant gap in the current understanding of optimal fixation methods for the acetabular cup.

The primary aim of our study was to assess the risk of all-cause cup revision in patients aged 65 years or older undergoing THA with either cemented or uncemented acetabular components. Secondary aims were to assess risks of cup revision due to dislocation, aseptic loosening, and other causes. Additionally, we aimed to analyze the risk of all-cause cup and/or stem revision.

## Methods

### Study design

We conducted a registry-based cohort study, utilizing prospectively collected data from the Nordic Arthroplasty Register Association (NARA) database. This study adheres to the Strengthening the Reporting of Observational Studies in Epidemiology (STROBE) guidelines [10].

### Data source

The NARA database was established in 2007 to facilitate collaborative research using standardized and harmonized data from arthroplasty registries across Denmark, Norway, Sweden, and Finland (which joined the collaboration in 2010) [11]. The coverage of hospitals reporting to the national arthroplasty registry is 100%, ensuring that all orthopedic departments, including private hospitals, systematically report procedures. The completeness of primary THA procedures in the national arthroplasty registries ranges during the study period from 90% to 98%, providing a highly reliable data source across participating countries. In the Nordic arthroplasty registers, revision completeness has generally been reported at around 85%–94%, although recent Swedish annual reports show hip-revision completeness up to 96% [12,13]. Each national registry undergoes regular validation through national patient registers to maintain data accuracy [12,14]. This collaboration benefits from the similarities in the healthcare systems, which are tax-funded, universally accessible, and collectively serve a population of approximately 27 million, providing a robust data source for research on THAs.

### Study population

All primary THAs performed between 1995 and 2018 were initially identified in NARA database.

Inclusion criteria: THA due to primary osteoarthritis, idiopathic femoral head necrosis/Perthes disease, developmental dysplasia of the hip, or epiphysiolysis capitis femoris, as THA for these childhood hip disorders has been shown to have a prognosis similar to THA for osteoarthritis [15,16].

Exclusion criteria: THA performed in patients younger than 65 years, or patients with inverse hybrid fixation, dual mobility cups, or resurfacing. Inverse hybrid fixation was excluded because it was not routinely used across all 4 countries and has become less common in recent years, potentially reducing the generalizability of our findings. Further, patients with metal-on-metal (MoM), ceramic-on-metal (CoM), and metal-on-ceramic (MoC) bearings were excluded due to well-known higher revision risk. Implants used fewer than 50 times in any participating country were also excluded to minimize the potential impact of learning curves, which could introduce variability unrelated to the implant itself. For individuals undergoing bilateral procedures, only the first recorded THA was included to ensure independent observations. Additionally, cases with missing information on cup fixation, missing data on potential confounders, or negative or no implant survival time were excluded. Patients had at least 2 years of follow-up and were followed from the date of primary surgery until revision, death, emigration, or end of study (December 31, 2020), whichever occurred first.

### Outcomes

The primary outcome was all-cause cup revision. Cup revision was defined as a surgical intervention involving any exchange or removal of the acetabular cup. Secondary outcomes included cause-specific cup revisions due to dislocation, aseptic loosening, and other causes. Additionally, all-cause cup and/or stem revision was investigated.

### Statistics

All statistical analyses were conducted based on a previously published statistical analysis plan [17]. Demographics are presented as frequencies or medians with interquartile ranges (IQRs). Risks of cup revision and any revision (cup and/or stem) were analyzed using Cox regression and reported as crude and adjusted hazard ratios (HR) with corresponding 95% confidence intervals (CI). The minimal adjustment set was defined using a directed acyclic graph: age group, sex, femoral head size, period of surgery (1995–2000, 2001–2006, 2007–2012, and 2013–2018), and nation. Risks of cause-specific cup revision were analyzed using Fine and Gray regression with death and other revision causes treated as competing risks. In order to meet the assumption of proportional hazards, all analyses were split into 3 time periods with proportional hazards 0–1 (first year), 1–6 (medium-term), and 6–26 years (long-term), which were identified using log–log plots and Schoenfeld residuals.

Prespecified analyses were stratified by sex and age group (65–69, 70–74, 75–79, and ≥ 80 years). Sensitivity analyses for cup and any revision were performed using Fine and Gray regression with death as a competing risk. Additionally, risk of cup revision was analyzed for patients with highly cross-linked polyethylene bearings (XLPE). Finally, risk of cup revision was analyzed for patients operated on with a posterior approach as a post hoc analysis not described in the statistical analysis plan.

In all regression models the uncemented cup was used as reference. The crude cumulative incidence functions were estimated using the Kaplan–Meier estimator and the Aalen–Johansen estimator when dealing with competing risks. Data were analyzed and plotted using Stata 18.0 BE (StataCorp, College Station, TX, USA).

### Ethics, registration, data sharing, use of AI, funding, and disclosures

This study was conducted in accordance with the ethical standards of the Declaration of Helsinki. Ethical approval was obtained from the appropriate regional Ethics Committees in Denmark, Norway, Sweden, and Finland: the Swedish Ethical Review Authority (Dnr: 1184-18/2019-00812), the Finnish National Institute of Health and Welfare (Dnro THL/2246/5.05.00/2019), the Norwegian Data Inspectorate (ref 24.1.2017: 16/01622-3/CDG and Ethical approval 2015/880/REK Vest), and the Danish Data Protection Agency (jr.nr.: 1-16-02-54-17). As this was a registry-based study using anonymized data, informed consent was not required.

This study was pre-registered with MedRxiv on May 27, 2024 (doi: 10.1101/2024.05.26.24307935).

Microsoft Copilot was used for language editing, including identifying dictation and typographical errors and improving concision to meet word limits. All suggested changes were reviewed and approved by the authors.

This study did not receive any external funding. CV received travel expenses from Stryker, which were paid to the institution and had no relevance to the present study. All authors declare no potential conflicts of interest with respect to the research, authorship, and/or publication of this article. Complete disclosure of interest forms according to ICMJE are available on the article page, doi: 10.2340/17453674.2026.46937

## Results

715,780 primary THAs were performed during the inclusion period. Most patients were excluded because they were younger than 65 years (n = 238,965), had a second THA during the study period (n = 61,432), or received implants used fewer than 50 times (n = 31,346) (Figure 1). The analysis population consisted of 310,515 patients, of whom 62% (n = 193,331) were women. Women accounted for 65% (n = 133,574) of the cemented cup group and 57% (n = 59,757) of the uncemented cup group. Patients receiving cemented cups were generally older than those receiving uncemented cups. From 1995 to 2006, cemented cups were used more frequently than uncemented cups, whereas uncemented cups were used more frequently between 2013 and 2018. Overall, 77% (n = 239,139) of all stems were cemented, whereas 32% (n = 32,828) of stems were cemented in the uncemented cup group. All stems were cemented in the cemented cup group because inverse hybrid THAs were excluded (Table 1). Median follow-up was 8.7 years (IQR 5.1–12.9) for cemented cups and 6.0 years (IQR 3.6–9.3) for uncemented cups.

**Figure 1:**
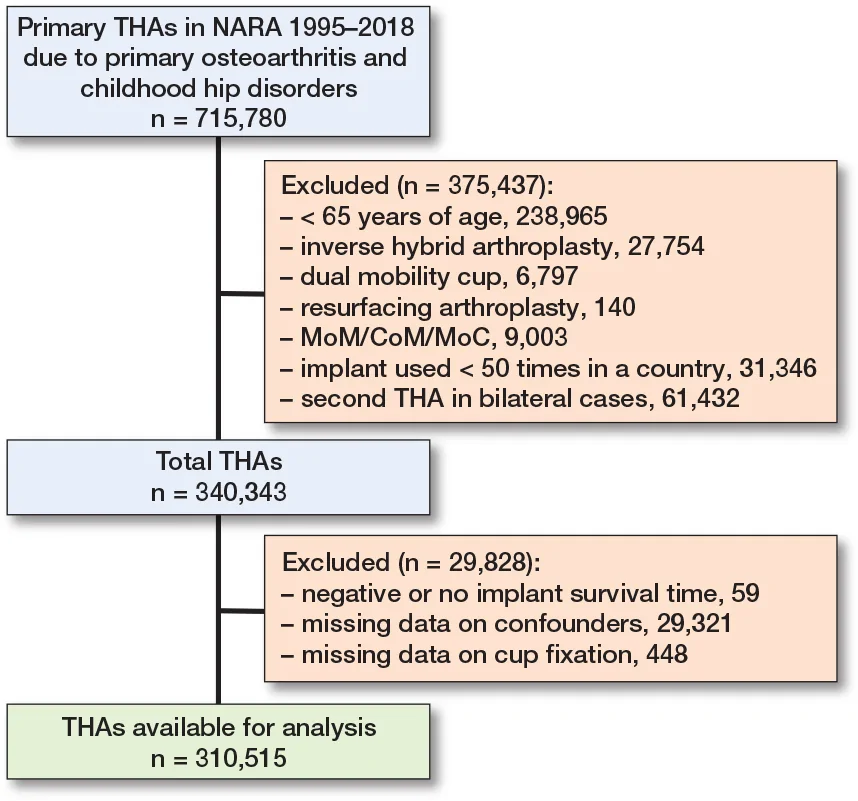
Flowchart of the study population.

**Table 1:** Patient characteristics and demographic data. Values are count (%) unless otherwise specified

Factor	Total n = 310,515	Cemented cup n = 206,311	Uncemented cup n = 104,204
Sex			
Women	193,331 (62)	133,574 (65)	59,757 (57)
Men	117,184 (38)	72,737 (35)	44,447 (43)
Age group			
65–69	85,057 (27)	46,058 (22)	38,999 (37)
70–74	89,397 (29)	58,462 (28)	30,935 (30)
75–79	75,020 (24)	54,649 (26)	20,371 (20)
≥ 80	61,041 (20)	47,142 (23)	13,899 (13)
Diagnosis			
Primary osteoarthrosis	300,285 (97)	199,855 (97)	100,430 (96)
Childhood hip disorders	10,230 (3.3)	6,456 (3.1)	3,774 (3.6)
Follow-up years, median (IQR)	7.7 (4.4–11.8)	8.7 (5.1–12.9)	6.0 (3.6–9.3)
Femoral fixation			
Cemented	239,139 (77)	206,311 (100)	32,828 (32)
Uncemented	71,376 (23)	0 (0.0)	71,376 (68)
Femoral head size, mm			
0–22	19,071 (6.1)	18,565 (9.0)	506 (0.5)
23–28	137,523 (44)	115,624 (56)	21,899 (21)
29–32	95,963 (31)	66,632 (32)	29,331 (28)
33–36	56,211 (18)	5,389 (2.6)	50,822 (49)
37–81	1,747 (0.6)	101 (0.0)	1,646 (1.6)
Bearing material ^**a**^			
PE ^**b**^	76,122 (43)	60,917 (77)	15,205 (16)
XLPE ^**c**^	95,024 (54)	18,375 (23)	76,649 (78)
Other (metal, ceramic, etc.)	5,864 (3.3)	4 (0.0)	5,860 (6.0)
Missing	133,505 (N/A)	127,015 (N/A)	6,490 (N/A)
Surgical approach			
Posterior approach	172,281 (55)	97,767 (47)	74,514 (72)
Other	135,697 (44)	107,339 (52)	28,358 (27)
Missing	2,537 (0.8)	1,205 (0.6)	1,332 (1.3)
Year of surgery			
1995–2000	38,556 (12)	32,976 (16)	5,580 (5.4)
2001–2006	80,559 (26)	67,056 (33)	13,503 (13)
2007–2012	85,540 (28)	57,471 (28)	28,069 (27)
2013–2018	105,860 (34)	48,808 (24)	57,052 (55)
Country			
Denmark	73,701 (24)	23,160 (11)	50,541 (49)
Norway	64,961 (21)	51,869 (25)	13,092 (13)
Sweden	123,348 (40)	112,281 (54)	11,067 (11)
Finland	48,505 (16)	19,001 (9.2)	29,504 (28)

a Proportion of complete cases.

b Polyethylene.

c Highly cross-linked polyethylene.

### Risk of all-cause cup revision

In the first year after surgery, the adjusted hazard ratio (aHR) indicated a lower risk of revision for cemented cups compared with uncemented cups (aHR 0.47, CI 0.41–0.53). From 6 years onward, cemented cups were associated with an increased risk of revision (aHR 1.91, CI 1.72–2.13). When stratified by sex and age group, the revision risk for cemented cups was lower in both men and women in all age groups during the first year after surgery. However, after 6 years in patients up to 79 years of age, the risk of cup revision for cemented cups was higher than for uncemented cups in both sexes. For patients aged 80 years or older, cup revision risk was lower with cemented cups during the first year in both sexes. After the first year, we found no evidence of an association between fixation method and cup revision (Figure 2, Table 2).

**Figure 2:**
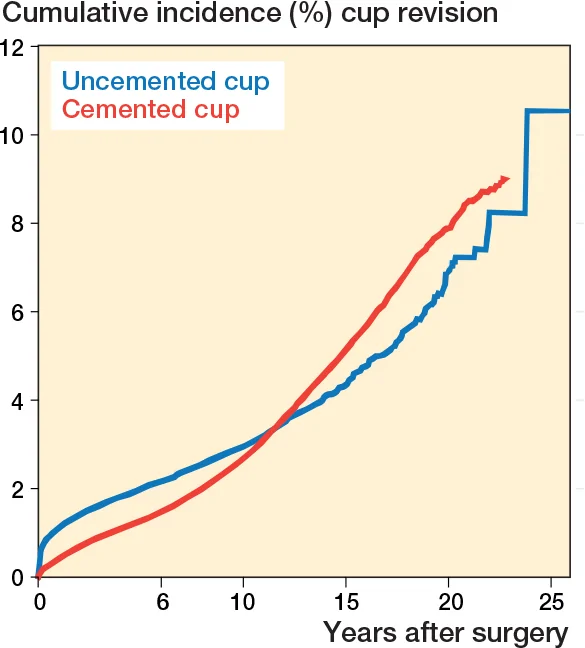
Cumulative incidence function of any cup revision.

**Table 2:** Hazard ratio (HR) of cemented cup revision compared with uncemented cup revision stratified by sex and age groups. Adjusted hazard ratios (aHRs) are adjusted for age group, sex, femoral head size, period of surgery, and nation

Sex	Age group	Follow-up, years	HR (CI)	aHR (CI)
All	All	0–1	0.36 (0.33–0.40)	0.47 (0.41–0.53)
		1–6	0.98 (0.91–1.07)	1.13 (1.01–1.26)
		6–26	1.53 (1.40–1.68)	1.91 (1.72–2.13)
Men	65–69	0–1	0.33 (0.25–0.43)	0.36 (0.25–0.52)
		1–6	1.25 (1.03–1.52)	1.00 (0.76–1.32)
		6–26	2.39 (1.99–2.87)	2.33 (1.87–2.90)
	70–74	0–1	0.32 (0.25–0.42)	0.33 (0.23–0.48)
		1–6	1.32 (1.04–1.67)	1.14 (0.82–1.59)
		6–26	2.04 (1.57–2.66)	1.69 (1.24–2.30)
	75–79	0–1	0.48 (0.36–0.63)	0.54 (0.36–0.81)
		1–6	1.53 (1.13–2.07)	1.53 (1.01–2.32)
		6–26	1.73 (1.10–2.72)	1.82 (1.04–3.17)
	≥ 80	0–1	0.31 (0.23–0.42)	0.34 (0.22–0.51)
		1–6	1.10 (0.72–1.68)	1.01 (0.58–1.75)
		6–26	0.96 (0.47–1.97)	1.25 (0.52–2.97)
Women	65–69	0–1	0.39 (0.30–0.49)	0.54 (0.38–0.75)
		1–6	1.07 (0.90–1.27)	1.50 (1.16–1.93)
		6–26	1.82 (1.55–2.14)	2.00 (1.66–2.42)
	70–74	0–1	0.43 (0.35–0.54)	0.71 (0.51–0.97)
		1–6	0.95 (0.79–1.15)	1.08 (0.83–1.39)
		6–26	1.36 (1.10–1.68)	1.63 (1.27–2.09)
	75–79	0–1	0.34 (0.26–0.44)	0.46 (0.32–0.67)
		1–6	0.75 (0.59–0.94)	0.82 (0.60–1.11)
		6–26	1.26 (0.92–1.74)	1.52 (1.04–2.23)
	≥ 80	0–1	0.30 (0.23–0.40)	0.51 (0.36–0.74)
		1–6	0.70 (0.53–0.92)	0.93 (0.65–1.33)
		6–26	0.83 (0.47–1.48)	0.84 (0.43–1.64)

CI = 95% confidence interval.

### Cause-specific risk of cup revision

2,593 uncemented and 6,026 cemented cups were revised. Among uncemented cup revisions, 44% were due to dislocation, compared with 23% of cemented cup revisions. Aseptic loosening accounted for 15% of uncemented cup revisions compared with 57% of cemented cup revisions. Other causes accounted for 41% of uncemented cup revisions and 20% of cemented cup revisions (Table 3).

**Table 3:** Revision causes by group of cup fixation. Values are count (%)

Revision cause	Total	Uncemented	Cemented
Cup revision due to any cause, n (%) ^**a**^	8,619	2,593	6,026
Cup revision due to dislocation	2,536 (29)	1,129 (44)	1,407 (23)
Cup revision due to aseptic loosening	3,811 (44)	397 (15)	3,414 (57)
Cup revision due to other causes	2,272 (26)	1,067 (41)	1,205 (20)
Any other revision ^**b**^	6,846	2,825	4,021

a Percentages refer to the proportion of each cause among all cup revisions, not the total number of primary THAs.

b Procedures not involving the acetabular cup, such as isolated stem revisions, implant removal without cup reimplantation, or revisions for infection, fracture, or mechanical failure where the cup was not revised.

#### Revision due to dislocation

Cemented cups showed a lower risk of cup revision due to dislocation compared with uncemented cups. For men, the risk of cup revision due to dislocation was lower for cemented cups during the first year, but during long-term follow-up the revision risk was similar between cemented and uncemented cups for men aged 65–79 years. For men aged 80 years or older, a reduced risk of cup revision was found for cemented cups during long-term follow-up. In women, reduced risk of cup revision was observed across all age groups and follow-up periods, except for long-term follow-up in the 65–69 age group, where no difference in revision risk was found between fixation methods (Figure 3, Table 4).

**Figure 3:**
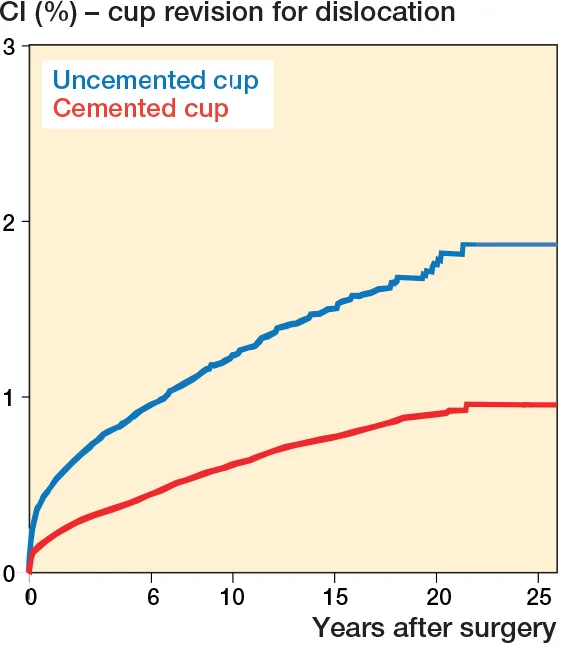
Cumulative incidence (CI) function of cup revision due to dislocation.

**Table 4:** Cause-specific hazard ratio (HR) of cemented cup revision compared with uncemented cup revision. Other revision causes included infection, periprosthetic fracture and mechanical failure. Adjusted hazard ratios (aHRs) are adjusted for age group, sex, femoral head size, period of surgery, and nation

Sex	Age group	Follow-up, years	Dislocation		Aseptic loosening		Other	
			HR (CI)	aHR (CI)	HR (CI)	aHR (CI)	HR (CI)	aHR (CI)
All	All	0–1	0.41 (0.36–0.46)	0.41 (0.34–0.49)	0.64 (0.48–0.85)	0.97 (0.61–1.54)	0.29 (0.25–0.33)	0.45 (0.36–0.55)
		1–6	0.52 (0.46–0.60)	0.53 (0.45–0.64)	2.57 (2.17–3.05)	3.71 (2.94–4.67)	0.80 (0.70–0.92)	0.82 (0.67–1.00)
		6–26	0.65 (0.56–0.76)	0.68 (0.55–0.84)	3.97 (3.38–4.67)	4.79 (3.98–5.75)	0.46 (0.39–0.55)	0.57 (0.45–0.72)
Men	65–69	0–1	0.34 (0.21–0.54)	0.20 (0.12–0.36)	0.89 (0.36–2.19)	1.21 (0.25–5.79)	0.28 (0.19–0.40)	0.45 (0.26–0.76)
		1–6	0.53 (0.36–0.78)	0.50 (0.29–0.86)	4.41 (2.85–6.82)	3.82 (2.30–6.34)	0.88 (0.64–1.21)	0.61 (0.38–0.98)
		6–26	0.95 (0.62–1.46)	1.03 (0.60–1.78)	6.10 (4.47–8.33)	4.96 (3.49–7.04)	0.65 (0.46–0.90)	0.61 (0.38–0.98)
	70–74	0–1	0.48 (0.32–0.73)	0.48 (0.28–0.81)	0.31 (0.09–1.04)	0.57 (0.15–2.16)	0.24 (0.16–0.34)	0.24 (0.15–0.39)
		1–6	0.54 (0.35–0.82)	0.42 (0.25–0.71)	3.01 (1.90–4.79)	2.71 (1.47–5.00)	1.26 (0.84–1.90)	1.21 (0.68–2.15)
		6–26	0.62 (0.37–1.05)	0.61 (0.32–1.16)	4.98 (3.20–7.73)	3.60 (2.20–5.90)	0.82 (0.50–1.34)	0.57 (0.30–1.08)
	75–79	0–1	0.44 (0.29–0.67)	0.33 (0.18–0.60)	2.18 (0.63–7.53)	7.00 (1.35–36.2)	0.42 (0.29–0.62)	0.60 (0.33–1.09)
		1–6	0.75 (0.46–1.21)	0.80 (0.41–1.55)	3.28 (1.64–6.56)	3.92 (1.47–10.4)	1.72 (1.05–2.84)	1.51 (0.90–2.54)
		6–26	0.45 (0.20–1.00)	0.41 (0.11–1.59)	2.86 (1.51–5.44)	2.72 (1.29–5.73)	3.16 (0.77–12.9)	3.66 (0.35–37.8)
	≥ 80	0–1	0.37 (0.24–0.56)	0.42 (0.25–0.72)	1.18 (0.24–5.66)	0.80 (0.14–4.73)	0.22 (0.14–0.35)	0.20 (0.09–0.45)
		1–6	0.59 (0.29–1.20)	0.77 (0.29–2.04)	2.21 (0.87–5.63)	1.76 (0.69–4.46)	1.15 (0.57–2.32)	0.81 (0.36–1.85)
		6–26	0.31 (0.11–0.84)	0.08 (0.03–0.28)	N/A	N/A	0.70 (0.15–3.23)	1.22 (0.29–5.05)
Women	65–69	0–1	0.40 (0.28–0.57)	0.41 (0.23–0.74)	0.53 (0.30–0.95)	0.78 (0.33–1.82)	0.33 (0.22–0.48)	0.60 (0.31–1.16)
		1–6	0.60 (0.45–0.80)	0.61 (0.40–0.92)	3.15 (2.24–4.45)	6.03 (3.62–10.05)	0.61 (0.44–0.85)	0.85 (0.51–1.41)
		6–26	0.93 (0.69–1.26)	0.93 (0.63–1.36)	4.94 (3.72–6.55)	4.80 (3.51–6.58)	0.38 (0.27–0.53)	0.39 (0.25–0.61)
	70–74	0–1	0.38 (0.27–0.52)	0.49 (0.30–0.80)	0.73 (0.39–1.38)	1.72 (0.52–5.62)	0.43 (0.31–0.62)	0.84 (0.52–1.37)
		1–6	0.57 (0.42–0.76)	0.64 (0.43–0.96)	2.58 (1.70–3.90)	3.81 (2.23–6.53)	0.72 (0.51–1.02)	0.57 (0.36–0.89)
		6–26	0.61 (0.44–0.84)	0.65 (0.43–0.99)	4.36 (2.84–6.69)	4.42 (2.76–7.08)	0.50 (0.32–0.77)	0.63 (0.35–1.11)
	75–79	0–1	0.30 (0.21–0.44)	0.35 (0.21–0.57)	0.67 (0.27–1.67)	1.08 (0.20–5.74)	0.34 (0.22–0.51)	0.59 (0.30–1.16)
		1–6	0.42 (0.30–0.60)	0.39 (0.25–0.62)	1.44 (0.92–2.26)	1.71 (0.94–3.12)	0.84 (0.53–1.35)	0.92 (0.48–1.77)
		6–26	0.65 (0.41–1.04)	0.52 (0.29–0.94)	3.93 (2.01–7.67)	4.35 (2.07–9.15)	0.51 (0.25–1.05)	0.86 (0.35–2.14)
	≥ 80	0–1	0.40 (0.28–0.56)	0.57 (0.36–0.92)	0.34 (0.16–0.73)	0.45 (0.14–1.46)	0.17 (0.10–0.28)	0.44 (0.23–0.85)
		1–6	0.38 (0.26–0.55)	0.40 (0.25–0.64)	2.74 (1.27–5.94)	4.68 (1.87–11.7)	0.72 (0.40–1.30)	1.08 (0.53–2.23)
		6–26	0.37 (0.19–0.74)	0.40 (0.16–0.99)	N/A	N/A	0.81 (0.18–3.55)	0.40 (0.07–2.16)

CI = 95% confidence interval.

N/A = no patients with cup revision due to aseptic loosening in the control group.

#### Cup revision due to aseptic loosening

The risk of cup revision due to aseptic loosening was higher for cemented compared with uncemented cups in the medium and long term across both sexes and all age groups (Table 4, Figure 4).

**Figure 4:**
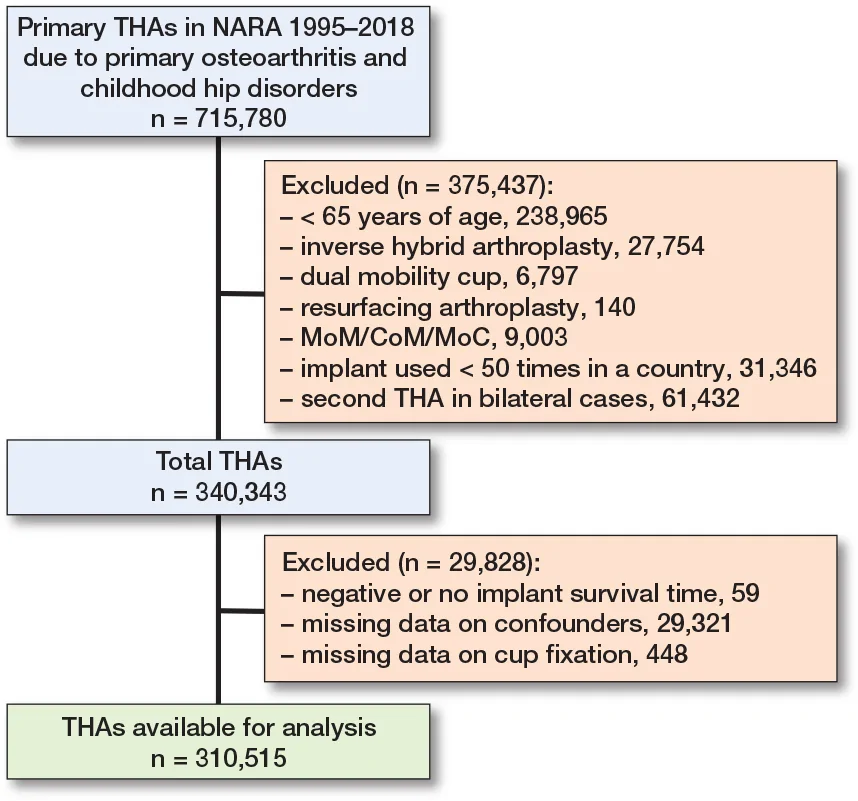
Cumulative incidence (CI) function of cup revision due to aseptic loosening.

#### Revision due to other causes

The revision risk due to other causes was lower or similar for cemented compared with uncemented cups across both sexes and all age groups (see Table 4).

### Risk of all-cause cup and/or stem revision

Patients with cemented cups consistently demonstrated significantly decreased risk of revision across all patient groups (aHR 0.61, CI 0.56–0.66) in the first year postoperatively. In men, no difference in risk of revision was found across any other follow-up periods except for men aged 65–69 years, where cemented cups resulted in an increased revision risk in the long-term follow-up (aHR 1.60, CI 1.36–1.88). Among women aged 65–69 years, cemented cups resulted in increased revision during long-term follow-up but for women aged 80 years or older, those with a cemented cup had a reduced revision risk in the short and long term, while no difference was observed in the medium term (Figure 5, Table 5).

**Figure 5:**
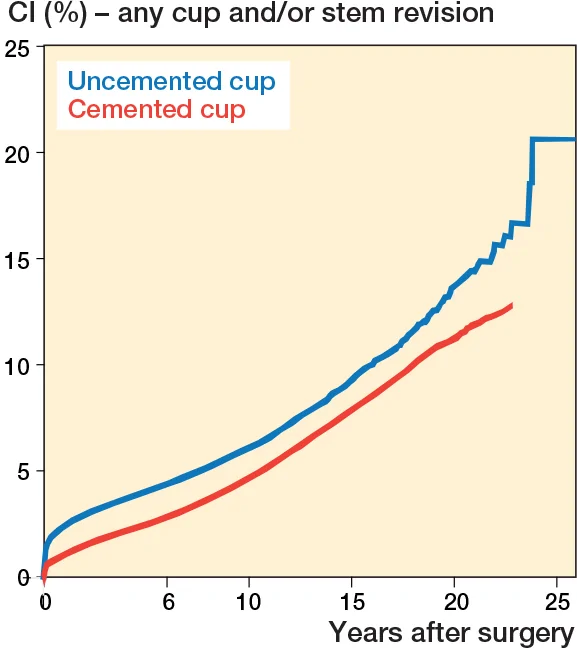
Cumulative incidence (CI) function of any cup and/or stem revision.

**Table 5:** Hazard ratio (HR) of any revision (cup, stem, or both) when cemented cup is used compared with uncemented cup. Adjusted hazard ratios (aHRs) are adjusted for age group, sex, femoral head size, period of surgery, and nation

Sex	Age group	Follow-up, years	HR (CI)	aHR (CI)
All	All	0–1	0.44 (0.41–0.46)	0.61 (0.56–0.66)
		1–6	0.87 (0.82–0.92)	0.99 (0.91–1.07)
		6–26	1.01 (0.95–1.07)	1.20 (1.12–1.30)
Men	65–69	0–1	0.44 (0.37–0.52)	0.59 (0.47–0.74)
		1–6	1.06 (0.92–1.24)	0.90 (0.73–1.11)
		6–26	1.57 (1.37–1.80)	1.60 (1.36–1.88)
	70–74	0–1	0.47 (0.40–0.56)	0.59 (0.47–0.75)
		1–6	1.07 (0.91–1.26)	0.97 (0.78–1.22)
		6–26	1.15 (0.97–1.37)	1.03 (0.84–1.27)
	75–79	0–1	0.46 (0.39–0.54)	0.57 (0.45–0.73)
		1–6	1.25 (1.01–1.54)	1.30 (0.97–1.75)
		6–26	0.92 (0.70–1.21)	0.92 (0.65–1.29)
	≥ 80	0–1	0.55 (0.45–0.67)	0.73 (0.55–0.98)
		1–6	0.93 (0.70–1.22)	0.85 (0.59–1.23)
		6–26	0.68 (0.43–1.06)	0.78 (0.45–1.37)
Women	65–69	0–1	0.35 (0.30–0.41)	0.55 (0.43–0.69)
		1–6	0.90 (0.79–1.03)	1.08 (0.89–1.31)
		6–26	1.21 (1.08–1.37)	1.37 (1.19–1.58)
	70–74	0–1	0.45 (0.38–0.52)	0.74 (0.60–0.91)
		1–6	0.85 (0.74–0.98)	0.98 (0.81–1.18)
		6–26	0.97 (0.83–1.13)	1.09 (0.92–1.30)
	75–79	0–1	0.40 (0.34–0.47)	0.60 (0.48–0.75)
		1–6	0.78 (0.65–0.93)	0.91 (0.73–1.14)
		6–26	0.75 (0.61–0.93)	0.81 (0.63–1.04)
	≥ 80	0–1	0.39 (0.33–0.47)	0.58 (0.47–0.73)
		1–6	0.60 (0.49–0.74)	0.79 (0.60–1.04)
		6–26	0.61 (0.43–0.86)	0.56 (0.38–0.85)

CI = 95% confidence interval.

### Risk of all-cause cup revision in patients with XLPE liners

XLPE was used in 18,375 (23% of complete cases) cemented cups and 76,649 (78% of complete cases) uncemented cups. Overall, cemented XLPE cups showed decreased risk of cup revision in the first year (aHR 0.56, CI 0.44–0.72), and in the medium term there was no difference in risk of cup revision regardless of fixation type. In the long term, cemented XLPE cups had a higher risk of cup revision compared with uncemented XLPE cups (aHR 1.86, CI 1.26–2.73) (Table 6).

**Table 6:** Hazard ratio (HR) of cemented cup revision compared with uncemented cup revision only including XLPE. Adjusted hazard ratios (aHRs) are adjusted for age group, sex, femoral head size, period of surgery, and nation

Sex	Age group	Follow-up, years	HR (CI)	aHR (CI)
All	All	0–1	0.43 (0.35–0.54)	0.56 (0.44–0.72)
		1–6	0.99 (0.82–1.19)	1.24 (0.99–1.54)
		6–26	1.35 (1.00–1.83)	1.86 (1.26–2.73)
Men	65–69	0–1	0.09 (0.01–0.66)	0.18 (0.02–1.37)
		1–6	1.36 (0.69–2.69)	1.52 (0.65–3.54)
		6–26	2.87 (1.24–6.64)	3.35 (1.00–11.2)
	70–74	0–1	0.32 (0.14–0.72)	0.31 (0.12–0.76)
		1–6	0.86 (0.43–1.73)	1.02 (0.46–2.26)
		6–26	1.14 (0.45–2.84)	0.92 (0.30–2.84)
	75–79	0–1	0.63 (0.35–1.12)	0.75 (0.39–1.46)
		1–6	2.19 (1.28–3.76)	2.12 (1.08–4.18)
		6–26	1.56 (0.46–5.34)	3.47 (0.72–16.8)
	≥ 80	0–1	0.31 (0.16–0.62)	0.29 (0.13–0.62)
		1–6	1.03 (0.42–2.51)	0.78 (0.27–2.19)
		6–26	0.79 (0.19–3.17)	1.34 (0.29–6.19)
Women	65–69	0–1	0.49 (0.25–0.96)	0.73 (0.34–1.57)
		1–6	0.90 (0.54–1.48)	1.47 (0.79–2.74)
		6–26	2.16 (1.10–4.22)	2.12 (0.83–5.43)
	70–74	0–1	0.52 (0.32–0.86)	0.74 (0.42–1.30)
		1–6	1.03 (0.70–1.52)	1.32 (0.84–2.07)
		6–26	1.65 (0.91–2.99)	2.49 (1.22–5.07)
	75–79	0–1	0.43 (0.25–0.74)	0.56 (0.30–1.03)
		1–6	0.82 (0.52–1.29)	0.92 (0.54–1.56)
		6–26	1.11 (0.50–2.47)	1.52 (0.60–3.81)
	≥ 80	0–1	0.47 (0.29–0.75)	0.61 (0.35–1.06)
		1–6	0.92 (0.54–1.56)	1.07 (0.56–2.03)
		6–26	N/A	N/A

CI = 95% confidence interval.

N/A = No patients with cup revision in the exposure group.

### Risk of all-cause cup revision with posterior surgical approach

To evaluate potential confounding from surgical approach, we performed a sensitivity analysis restricted to THAs performed using the posterior approach (cemented cups: n = 97,767, uncemented cups: n = 74,514). Results were consistent with the main analysis: cemented cups had a lower risk of cup revision in the short term (aHR 0.55, CI 0.47–0.64), no significant difference in the medium term (aHR 1.12, CI 0.98–1.28), and a higher risk of cup revision in the long term (aHR 1.81, CI 1.58–2.07). When stratifying for sex and age, analyses confirmed similar patterns, particularly increased long-term revision risks for cemented cups in younger men aged 65–69 (aHR 2.24, CI 1.68–2.99) (Table 7).

**Table 7:** Hazard ratio (HR) of cemented cup revision compared with uncemented cup revision when operated on through a posterior approach. Adjusted hazard ratios (aHRs) are adjusted for age group, sex, femoral head size, period of surgery, and nation

Sex	Age group	Follow-up, years	HR (CI)	aHR (CI)
All	All	0–1	0.35 (0.32–0.39)	0.55 (0.47–0.64)
		1–6	0.91 (0.82–1.00)	1.12 (0.98–1.28)
		6–26	1.47 (1.31–1.64)	1.81 (1.58–2.07)
Men	65–69	0–1	0.33 (0.23–0.46)	0.49 (0.30–0.81)
		1–6	1.08 (0.84–1.39)	0.92 (0.64–1.33)
		6–26	2.15 (1.71–2.71)	2.24 (1.68–2.99)
	70–74	0–1	0.29 (0.20–0.40)	0.35 (0.22–0.56)
		1–6	0.98 (0.74–1.30)	0.94 (0.63–1.39)
		6–26	2.10 (1.49–2.97)	1.88 (1.24–2.84)
	75–79	0–1	0.37 (0.26–0.53)	0.50 (0.30–0.83)
		1–6	1.18 (0.83–1.68)	1.46 (0.89–2.38)
		6–26	1.54 (0.93–2.55)	1.40 (0.76–2.59)
	≥ 80	0–1	0.24 (0.16–0.35)	0.27 (0.16–0.46)
		1–6	0.84 (0.51–1.39)	0.87 (0.45–1.68)
		6–26	0.70 (0.31–1.59)	0.77 (0.29–2.03)
Women	65–69	0–1	0.41 (0.31–0.55)	0.69 (0.45–1.05)
		1–6	0.99 (0.80–1.23)	1.78 (1.31–2.43)
		6–26	1.70 (1.39–2.08)	1.98 (1.55–2.52)
	70–74	0–1	0.45 (0.35–0.59)	0.95 (0.66–1.37)
		1–6	0.91 (0.73–1.14)	1.07 (0.79–1.46)
		6–26	1.33 (1.02–1.73)	1.62 (1.18–2.23)
	75–79	0–1	0.33 (0.24–0.45)	0.55 (0.35–0.85)
		1–6	0.89 (0.67–1.18)	1.11 (0.76–1.61)
		6–26	1.13 (0.78–1.64)	1.29 (0.83–2.01)
	≥ 80	0–1	0.33 (0.24–0.44)	0.56 (0.38–0.83)
		1–6	0.67 (0.49–0.91)	0.80 (0.54–1.20)
		6–26	0.65 (0.34–1.26)	0.73 (0.33–1.60)

CI = 95% confidence interval.

### Sensitivity analyses with death as a competing risk

Sensitivity analyses were performed for all-cause cup revision and all-cause cup and/or stem revision using Fine and Gray regression to account for death as a competing risk and the overall findings remained consistent with the main analyses.

## Discussion

We aimed to estimate the risk of revision of cemented vs uncemented cups in elderly patients undergoing primary THA. We showed that cemented acetabular cups demonstrated a lower risk of revision during the first year postoperatively compared with uncemented cups. However, the long-term (6–26 years) revision risk of cemented cups was increased. Among patients aged 80 years or older, cemented cups were associated with a lower first-year cup revision risk; after the first year, we found no evidence of an association between fixation method and cup revision. Cause-specific analyses revealed that cemented cups were associated with a lower risk of revision due to dislocation across all time periods, but a higher risk of revision due to aseptic loosening.

Several studies have highlighted the early benefits of cemented acetabular fixation in reducing the risk of early dislocation, possibly explained by the early stability provided by cement fixation [7,18-20]. Our results align with these findings, as we observed a lower risk of revision due to dislocation associated with cemented cups across all follow-up periods, particularly benefiting elderly patients who may have reduced bone quality. This may be explained by more optimal cup positioning during surgery, particularly in elderly patients with compromised bone quality.

Not all studies agree with our findings, particularly regarding the long-term performance of cemented cups. A meta-analysis by Toossi et al. reported better long-term survivorship for cemented compared with uncemented acetabular components [21]. The discrepancy may be due to differences in study populations, implant design, and follow-up methods. Additionally, many of the studies included in the meta-analysis were not based on nationwide registry data, which may limit their comparability to our population-level dataset.

After the initial postoperative period, the biological fixation of uncemented cups becomes reliable and strong, allowing these cups to perform better in the long term compared with cemented cups. This aligns with the findings of Van Praet and Mulier, who reported a higher revision rate for cemented cups due to aseptic loosening [7]. Similarly, Hooper et al. observed that cemented cup fixation resulted in increased rates of aseptic loosening in elderly patients [22]. These findings are consistent with our study, where uncemented cups showed a lower risk of revision after the 6-year mark, particularly for aseptic loosening. Increased revision risk due to aseptic loosening of the cemented cups may be due to the continuous bone loss and resorption in the bone–cement interface. Polyethylene type may also have influenced the magnitude of the long-term difference, as XLPE was substantially more common in uncemented than cemented cups (78% vs 23% of complete cases). However, the XLPE-only analysis showed the same overall pattern, suggesting that differential polyethylene use did not fully explain the main findings. Fixation choice should remain individualized according to patient-specific factors such as age, sex, bone quality, and expected longevity.

At 6–26 years, cemented cups were associated with a higher cup revision risk in patients aged 65–79 years. Among patients aged 80 years or older, we found no evidence of an association between fixation method and cup revision after the first year, although confidence intervals were wide. These findings have not previously been shown in large studies.

### Strengths

A major strength of this study is the inclusion of a large real-world dataset from Nordic national arthroplasty registries with high completeness for both primary and revision procedures [12]. This supports the internal validity of the study and the generalizability of the findings.

### Limitations

Confounding by indication may have influenced the observed associations, as the choice between cemented and uncemented fixation is affected by factors not captured in the registry. These may include markers of poor bone quality, such as diagnosed osteoporosis, a previous fragility fracture, or prior glucocorticoid treatment, which may have led surgeons to use cemented cups more often than uncemented cups. Although uncemented cups are generally used in patients with good bone quality, in some settings they may also be preferred in patients with compromised bone stock when additional screw fixation is used.

Other confounders including patient comorbidity, activity level, and cup positioning could also affect comparability between groups. Finally, unmeasured confounding may persist due to variables such as surgeon experience, hospital volume, and surgical technique, all of which are known to influence THA outcomes but are not captured in the NARA dataset [23–25].

Femoral head size was included in all adjusted models; although smaller heads were more common with cemented cups (see Table 1), the unadjusted risk of cup revision due to dislocation was lower with cemented cups. Cement brand and acetabular cementing technique are not available in the NARA dataset, so the significance of these variables cannot be investigated.

The age- and sex-stratified analyses were prespecified. Confidence intervals were wide in some strata, particularly after the first year among patients aged 80 years or older.

### Conclusions

Cemented acetabular cups were associated with a reduced risk of cup revision during the first year across all age groups. At 6–26 years, cemented cups were associated with a higher cup revision risk in patients aged 65–79 years, consistent with the overall analysis. Among patients aged 80 years or older, we found no evidence of an association between fixation method and cup revision after the first year. Cemented cups were associated with a lower risk of cup revision due to dislocation.

## Notes

SO conceived the study, and MM contributed to formulating the research question. CV, SO, SK, and RL developed the study design and methodology. CV also contributed to the critical review and refinement of the study design and methods. SK performed the statistical analyses. RL wrote the first draft of the manuscript and revised it in collaboration with the coauthors. All authors critically reviewed the methods and manuscript. CV and SO jointly supervised the study.

Handling co-editors: Taco Gosens and Philippe Wagner

*Acta* thanks Sarunas Tarasevicius and Wierd P. Zijlstra for help with peer review of this manuscript.
